# Drivers of high-cost persistence in rural China: A population-based retrospective study

**DOI:** 10.3389/fpubh.2022.988664

**Published:** 2022-12-06

**Authors:** Chenzhou Wang, Shan Lu, Yan Zhang

**Affiliations:** ^1^School of Medicine and Health Management, Tongji Medical College, Huazhong University of Science and Technology, Wuhan, China; ^2^Research Centre for Rural Health Service, Key Research Institute of Humanities and Social Sciences of Hubei Provincial Department of Education, Wuhan, China

**Keywords:** high-cost patients, high-cost persistence, persistently high-cost patients, transition probability matrix, rural China

## Abstract

**Purpose:**

High-cost patients account for over 70% of total health expenditures in rural China and have become a key focus of health insurers. Persistently high-cost patients constitute a substantial proportion of medical resources. Hence, exploring high-cost persistence (HCP) and what drives it is considered meaningful and necessary.

**Patients and methods:**

A population-based retrospective study was carried out. The annual healthcare utilization data of Dangyang New Rural Cooperative Medical Scheme from 2012 to 2017 were analyzed. Patients in the top 10% of spending in a given year were considered high-cost patients. Persistence level was estimated using Markov matrices. A total of 19,405 patients categorized as high-cost patients in 2016 were divided into two groups according to whether or not they kept high-cost status in 2017. Finally, a multilevel logistic regression model was used in examining the determinants of HCP.

**Results:**

On average, about 31.48% of high-cost patients each year still maintained high-cost status in the subsequent year from 2012 to 2017. The elderly (OR = 2.150), families with more non-labor members (OR = 2.307), families applying for subsistence allowances (OR = 1.245), and patients with blood and immune diseases (OR = 2.614) or malignant tumors (OR = 2.077) were more likely to maintain high-cost status. Hospitalization frequency was found to be a mediator.

**Conclusion:**

About one-third of high-cost patients in a given year had persistently high cost status in the subsequent year. Health status and family support were considered the main drivers of HCP. High inpatient service utilization as a mediator was a prominent manifestation of persistently high-cost patients. The accurate identification of persistently high-cost patients is the basis for our management.

## Introduction

High-cost patients, constituting a small group of the population that generates most medical expenditures, have become a research object of widespread concern. Patients in a high-cost state may maintain this state in the following years (1–3). The concept of expenditure persistence has been refined into high-cost persistence (HCP), which refers to the probability that a patient will continue to be in a high-cost state (top 10% of annual medical expenditure distribution) within a certain time span (at least 2 years) (4, 5). Persistently high-cost patients who maintain a high-cost state within a certain time span are significant parts of high-cost patients. Heavy economic burden is imposed on high-cost patients, likely leading to catastrophic health expenditures (6). As for persistently high-cost patients, the financial burden is more serious.

However, studies on the level and drivers of HCP in China are limited. Some developed countries have explored HCP, including the level of HCP and expenditure composition of the persistently high-cost population (5). However, in China, the high-cost population is mainly analyzed in a single year, without exploring the persistence of time series and analyzing the characteristics. Therefore, the persistence level and characteristics of the persistently high-cost population in rural China are the research interest of this study. In terms of HCP level, panel data based on an entire population in a county are useful in obtaining overall HCP. In terms of influencing factors, high-cost status is usually related to age, race, health status, health insurance status, and income (7). Health status is prominently reflected in co-morbid illnesses, multiple chronic conditions, and mental illnesses (7). Meanwhile, the sample range of persistently high-cost patients is limited. Previous studies selected different sample domains, such as patients with certain diseases or specific insurance beneficiaries (8, 9). Furthermore, high-cost status is highly correlated with hospitalization. Our team's previous work in rural China confirmed this finding (10).

How to accurately identify persistently high-cost patients for targeted intervention should be considered by policymakers. The Chinese government has introduced relevant policies to alleviate economic burden on patients, for instance, Supplementary Medical Insurance for Major Illnesses and Healthy Targeted Poverty Alleviation Policy (Supplementary Table 1), which were implemented in 2015 and 2016, respectively. Although the implementation of these policies has alleviated the economic burden on some residents to a certain extent, the catastrophic health expenditure incidence of rural households had not decreased significantly from 2012 (18.42%) to 2018 (18.31%) (11). Target populations should be accurately identified for efficient and effective implementation. In terms of policy, the identification and management of persistently high-cost groups is critical but remains lacking.

This study aims to estimate the HCP level in rural China, analyze the characteristics of persistently high-cost patients, and identify influencing factors from a dynamic and continuous perspective.

## Materials and methods

### Study design and sample

We carried out a population-based retrospective study to explore HCP and its driving factors. The significant concept transition probability in the Markov chain was used in estimating HCP. The medical spending status of residents transferred between different years, which generated a transition probability among statuses. Our focus was the probability of high-cost patients with persistent high-cost status to estimate HCP. As for driving factors, we used the stepwise logistic regression model with transfer outcome as the dependent variable and then tested hospitalization as a mediating variable based on our hypotheses.

Data were obtained from Dangyang Data Source from 2012 to 2017. Dangyang, a typical rural area in Hubei, central China, had a rural population of 331,349 and a gross domestic product per capita of US$15,596.0 in 2017 (exchange rate in 2017: RMB¥6.75 to US$1.00), which was above the national average (US$9481.9). The sample county had 2 county hospitals, 18 township health centers, and 158 village clinics. The New Rural Cooperative Medical Scheme (NCMS) is the primary insurance program in rural China. It has been implemented since 2003, and the coverage rate reached more than 95% in 2012 (12). Nearly all Dangyang residents were enrolled in the NCMS from 2012 to 2017. When residents used their NCMS accounts to visit multilevel public medical institutions, service utilization records and consumption amounts were recorded in the NCMS database. The NCMS reimbursed residents at different rates depending on the type of patient service utilization (outpatient or inpatient) and the hierarchy of medical institutions (primary or county hospitals). The original NCMS data mainly included three parts: personal information, outpatient utilization information, and inpatient utilization information. According to individual ID and household ID, we combined all annual outpatient and inpatient records for each enrollee into a single summary record and established panel data from 2012 to 2017. We excluded deaths and beneficiaries who did not incur medical expenditures in the analyzed years. Data were sorted in descending order according to the annual medical expenditure in the record. The top 10% of the cases were high-cost patients. This study used the 2012–2017 medical service utilization data from the Dangyang NCMS database and mainly analyzed the high-cost patients' data from 2016 to 2017 (*n* = 19,405) to identify the driving factors.

### Measures

#### High-cost persistence and persistently high-cost patients

Patients in the top 10% of total annual individual spending were defined as high-cost patients. We selected this threshold on the basis of previous studies on high-cost populations (5, 13). HCP was designed to be estimated by high-cost transfer outcome which was interpreted as whether patients in the top 10% of the initial annual spending ranking remain high-cost patients within a period measured in years. For instance, when we analyzed the characteristics of HCP from 2016 to 2017, the transition outcome was whether high-cost patients in 2016 maintained their status by 2017. The high-cost patients in 2016 can be divided into two groups based on transfer outcome. The two groups were named persistently high-cost patients and transiently high-cost patients.

#### Associated factors of high-cost persistence

The independent variables included socio-demographic, socio-environmental, disease, and healthcare utilization variables (Supplementary Table 3), which were based on our data availability and previous studies on high-cost status. Socio-environmental variables were included because our previous analysis showed that the obvious distribution characteristics of the annual high-cost residents were due to the social environment. We extracted family codes from the NCMS to construct family structure variables; obtained residential address to form socio-environmental variables: terrain, township health center service capacity, and distance to county hospital; and grouped main diagnoses into clinically similar groups by integrating their associated ICD-10 codes (Supplementary Table 2). Healthcare utilization data were obtained from the initial year (2016).

According to the Anderson model, predisposing characteristics, enabling resources, need factors, and psychosocial factors are the determinants of healthcare use (14). On this basis, we established hypothesis 1: socio-demographic, socio-environmental, and disease factors would affect healthcare utilization. Previous studies have confirmed that inpatient service utilization is highly correlated with high-cost status (15). Hence, we established hypothesis 2: hospitalizations in the initial year would affect the HCP. Hypothesis 3 stated that socio-demographic, socio-environmental, and disease factors would affect the HCP. On the basis of hypotheses 1, 2, and 3, we constructed a mediation model and used hospitalization frequency as the mediator.

#### Statistical analysis

To estimate the HCP level, we used MATLAB 2019b to process the database and count the cases where high-cost patients in any year from 2012 to 2017 maintained their high-cost status in any of the subsequent years and then calculated the probability. The characteristics of the high-cost patients in the persistently and transiently groups were compared by conducting *t*-test and chi-square test. Independent variables were divided into four categories: socio-demographic, socio-environmental, disease, and healthcare utilization factors, which were successively included in the regression equations. On this basis, using the transfer outcome as the dependent variable (1 = persistently, 0 = transiently), we tested the stepwise binary logistic regression models, which passed the Hosmer-Lemeshow test (*p* ≥ 0.05). Then, to verify the hypotheses of the mediating effect, we used hospitalization frequency as the key mediating variable to test the path relationship. All data analyses were conducted in Stata 15.1.

### Ethics approval and consent to participate

This study was approved by the ethics committee of Tongji Medical College, Huazhong University of Science and Technology (IORG No: IORG0003571). The Research Ethics Committee Approval Form is available in Supplementary material. Our team obtained permission from the Dangyang Healthcare Security Administration to use the private data for this research through a signed cooperation agreement. Data acquired were kept anonymized.

## Results

### High-cost persistence level from 2012 to 2017

As shown in Table 1, the HCP within two consecutive years was ~31.48% (weighted average). The highest probability was obtained during the transfer from 2014 to 2015 (40.47%), and the lowest was obtained from 2016 to 2017 (19.71%). Notably, the transition probabilities were related to the length of interval time. Approximately 40.47% of residents with high-cost status in 2014 maintained high-cost status in 2015, but this probability gradually decreased over time. In 2017, only 15.11% maintained high-cost status.

**Table 1:** Transition probabilities of maintaining high-cost between years (%).

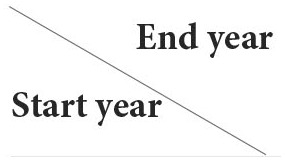	**2013**	**2014**	**2015**	**2016**	**2017**
2012	38.52	30.61	13.53	14.49	13.25
2013	-	34.38	15.45	14.94	12.06
2014	-	-	40.47	15.84	15.11
2015	-	-	-	25.34	18.72
2016	-	-	-	-	19.71

### Characteristic distribution of different high-cost groups

Table 2 shows significant differences (*P* < 0.001) between two groups for three demographic items: age, subsistence allowance, and burden population ratio. Specifically, compared with the transiently high-cost group, the persistently high-cost group had larger proportions of elderly people (50.11%) and families with subsistence allowances (27.03%). A higher burden population ratio (0.67) was a feature of the persistently high-cost group. In addition, no statistical differences in gender, terrain, distance to county hospital, and township health center service capacity were found between the groups (*P* > 0.05).

**Table 2:** Socio-demographic and socio-environmental characteristic distribution (*n* = 19,405).

**Characteristics^a^**	**Overall (*n* = 19,405)**	**Persistently high-cost (*n* = 1,776)**	**Transiently high-cost (*n* = 17,629)**	***T*/*F***	***P-*value**
Gender (n, %)					
Male	8,707 (44.87)	785 (44.20)	7,922 (44.94)	0.354^c^	0.552
Female	10,698 (55.13)	991 (55.80)	9,707 (55.06)		
Age (mean, SD)	48.47 (22.80)	56.85 (19.90)	47.63 (22.91)	18.342^b^	< 0.001
Age (*n*, %)					
≤ 5	1,507 (7.77)	66 (3.72)	1,441 (8.17)	248.462^c^	< 0.001
6~18	489 (2.52)	24 (1.35)	465 (2.64)		
19~40	3,935 (20.28)	227 (12.78)	3,708 (21.03)		
41~59	6,776 (34.92)	569 (32.04)	6,207 (35.21)		
≥60	6,698 (34.52)	890 (50.11)	5,808 (32.95)		
Burden population ratio (mean, SD)	0.56 (0.29)	0.67 (0.33)	0.55 (0.29)	13.855^b^	< 0.001
Subsistence allowances (*n*, %)					
Yes	3,371 (17.37)	480 (27.03)	2,891 (16.40)	126.964^c^	< 0.001
No	16,034 (82.63)	1,296 (72.97)	14,738 (83.60)		
Terrain (*n*, %)					
Plain	13,426 (69.19)	1,234 (69.48)	12,192 (69.16)	4.137^c^	0.388
Downland	2,469 (12.72)	231 (13.01)	2,238 (12.69)		
Hill	2,823 (14.55)	239 (13.46)	2,584 (14.66)		
Mountain	680 (3.50)	72 (4.05)	608 (3.45)		
Township health center service capacity (*n*, %)					
High	9,335 (48.11)	823 (46.34)	8,512 (48.30)	2.613^c^	0.271
Middle	6,326 (32.60)	604 (34.01)	5,722 (32.47)		
Low	3,737 (19.26)	349 (19.65)	3,388 (19.23)		
Distance to county hospital (mean, SD), km	15.67 (9.13)	15.86 (8.86)	15.65 (9.15)	0.946^b^	0.344

^a^The definitions of the variables are detailed in Supplementary Table 3.

^b^T value from pearson's chi-square test.

^c^F-value from ANOVA.

Table 3 shows significant differences (*P* < 0.001) in main disease and healthcare utilization. The persistently high-cost group had higher risks of malignant tumors (26.63%), blood and immune system diseases (12.78%), and infectious and parasitic diseases (14.70%). Moreover, the persistent group had a higher hospitalization utilization rate in the initial year. Residents in the persistently high-cost group had an average of 2.25 hospitalizations and 34.36 days per person, and often preferred county hospitals for treatment.

**Table 3:** Main disease and healthcare utilization characteristic distribution (*n* = 19,405).

**Characteristics^a^**	**Overall (*n* = 19,405)**	**Persistently high-cost (*n* = 1,776)**	**Transiently high-cost (*n* = 17,629)**	***T*/*F***	***P-*value**
Main disease (*n*, %)					
Infectious and parasitic	2,353 (12.13)	261 (14.70)	2,092 (11.87)	277.599^c^	< 0.001
Malignant tumor	3,934 (20.27)	473 (26.63)	3,461 (19.63)		
Blood and immune	1,404 (7.24)	227 (12.78)	1,177 (6.68)		
Endocrine and metabolic	2,278 (11.74)	170 (9.57)	2,108 (11.96)		
Nervous system and mental	1,147 (5.91)	141 (7.94)	1,006 (5.71)		
Circulatory system	1,443 (7.44)	119 (6.70)	1,324 (7.51)		
Respiratory system	2,570 (13.24)	195 (10.98)	2,375 (13.47)		
Genitourinary system	2,021 (10.41)	84 (4.73)	1,937 (10.99)		
Others	2,255 (11.62)	106 (5.97)	2,149 (12.19)		
Hospitalization frequency (mean, SD)	1.51 (1.43)	2.25 (2.44)	1.44 (1.26)	13.872^b^	< 0.001
Length of stay (mean, SD)	16.94 (27.53)	34.36 (57.91)	15.18 (21.52)	13.857^b^	< 0.001
Hospitalization frequency of institutions (mean, SD)					
Township Health Center	0.36 (0.87)	0.50 (1.09)	0.34 (0.84)	5.965^b^	< 0.001
County hospital	0.91 (1.17)	1.41 (2.28)	0.85 (0.98)	10.184^b^	< 0.001
Outside of the county	0.26 (0.68)	0.40 (0.96)	0.25 (0.64)	6.232^b^	< 0.001
Outpatient visits (mean, SD)	5.09 (7.49)	4.43 (7.50)	5.16 (7.49)	−3.880^b^	< 0.001

^a^The definitions of the variables are detailed in Supplementary Table 3.

^b^T-value from pearson's chi-square test.

^c^F-value from ANOVA.

### Determinants of high-cost persistence

Table 4 displays the stepwise binary logistic regression model estimation results. In general, age, family support, disease category, and healthcare utilization factors were identified as the major determinants of HCP, whereas the impact of socio-environmental factors was not significant. Middle-aged and elderly patients were more likely to maintain high-cost status. High-cost patients with fragile family structures or subsistence allowances had a higher probability to be persistent. High-cost patients with specific diseases were more likely to maintain a high-cost status, especially those with malignant tumors and blood and immune diseases. Inpatient service utilization and outpatient service utilization had opposite effects on HCP. The HCP increased with the total length of stay. Out-of-county hospitalization was a significant risk factor.

**Table 4:** Binary logistic regression models of high-cost persistence (*n* = 19,405).

**Characteristics^a^**	**Model I**	**Model II**	**Model III**
	**OR(SE)**	**OR(SE)**	**OR(SE)**
Gender (male)^b^
Female	1.057 (0.051)	1.117 (0.052)**	1.141 (0.054)**
Age (≤5)^b^
6~18	1.122 (0.245)	1.381 (0.247)	1.361 (0.249)
19~40	1.407 (0.145)**	1.915 (0.151)***	1.751 (0.154)***
41~59	1.961 (0.134) ***	1.972 (0.138) ***	1.946 (0.139) ***
≥60	2.265 (0.136)***	1.983 (0.139)***	2.150 (0.141)***
Burden population ratio	2.458 (0.111)***	2.417 (0.110)***	2.307 (0.112)***
Subsistence allowances (No)^b^
Yes	1.650 (0.059)***	1.578 (0.059)***	1.245 (0.063)***
Terrain (plain)^b^
Downland	1.027 (0.082)	1.022 (0.082)	0.982 (0.084)
Hill	0.884 (0.083)	0.876 (0.083)	0.854 (0.087)*
Mountain	1.103 (0.142)	1.101 (0.143)	1.009 (0.147)
Distance to county hospital	1.002 (0.003)	1.002 (0.003)	1.002 (0.003)
Township health center service capacity	0.954 (0.038)	0.945 (0.038)	0.917 (0.039)**
Main disease (others)^b^
Infectious and parasitic	-----	2.125 (0.122)***	1.626 (0.126)***
Malignant tumor	-----	2.342 (0.115)***	2.077 (0.116)***
Blood and immune	-----	2.933 (0.128)***	2.614 (0.131)***
Endocrine and metabolic	-----	1.490 (0.129)***	1.365 (0.129)**
Nervous system and mental	-----	2.454(0.136)***	1.151(0.161)
Circulatory system	-----	1.596 (0.140)***	1.470 (0.141)***
Respiratory system	-----	1.757 (0.126) ***	1.457 (0.127)***
Genitourinary system	-----	0.932 (0.160)	0.895 (0.161)
Hospitalization frequency	-----	-----	0.950 (0.059)
Hospitalization frequency in township	-----	-----	1.025 (0.062)
Hospitalization frequency in county	-----	-----	1.055 (0.062)
Hospitalization frequency outside of county	-----	-----	1.170 (0.064)**
Length of stay	-----	-----	1.012 (0.001)***
Outpatient visits	-----	-----	0.974 (0.004)***

^a^The definitions of the variables are detailed in Supplementary Table 3.

^b^stands for the reference of dummy variable.

***P < 0.01; **P < 0.05; *P < 0.1.

Based on the theoretical hypothesis that hospitalization is a mediating factor, the mediation effect models are shown in Table 5. Hospitalization frequency partially mediated the relationships between burden population ratio, subsistence allowance, main disease, and HCP, and the effect sizes were 6.14, 43.03, and 11.55%. This result indicated that these factors not only directly affected HCP but also affected it through hospitalization frequency. Although the capacity of township health centers had no direct effect on HCP, the capacity of township health centers influenced HCP through hospitalization, and hospitalization played a completely mediating effect.

**Table 5:** Effect of hospitalization frequency as a mediator factor (*n* = 19,405)^a^.

**Predictor (*X*)**	**Model *R^2^***	**Total effect (*c* path)**	**Direct effect (*c'* path)**	**Indirect effect (*****ab*** **path)**^**b**^	
				a*b	***95% BootCI***
Burden population ratio	0.039***	0.073 (0.008)***	0.068 (0.008)***	0.004	(0.002, 0.007)
Subsistence allowances	0.036***	0.0489 (0.006) ***	0.027 (0.006) ***	0.020	(0.021, 0.032)
Main disease	0.028***	−0.004 (0.001) ***	−0.004 (0.001) ***	−0.000	(−0.006, −0.002)
Township health center service capacity	0.027***	−0.003 (0.003)	−0.005 (0.003)	0.001	(0.001, 0.006)

^a^Mediator, hospitalization frequency; criterion, transfer outcome.

^b^Effect size = a*b/c.

***P < 0.01.

## Discussion

### A proportion of high-cost patients exhibit persistently high-cost status

We found that about one-third of patients in a given year maintained high-cost status in the subsequent year, continuing to incur high medical expenses. This proportion is consistent with the results of the 2008–2012 US statistics showing that 32% of the top 10% high-cost individuals in Veterans Affairs maintained high-cost status after 1 year (16). When the time interval was extended to 2 years or more, the persistence rate was reduced to about 15%, which was lower than the rates in some developed countries' studies. An analysis of Medicaid beneficiaries in the United States showed a 5-year high-cost persistent rate of 49.2%, and a survey of Canadian public medical insurance beneficiaries showed about 30% over 3 years (17, 18). This difference may be related to the population covered by different insurance services. In rural China, the NCMS nearly covered the entire population, whereas the databases of other relevant studies only covered the insurance beneficiaries of Medicare, Medicaid, and Veterans Affairs. These insurance services tend to target specific groups rather than the whole population. Medicare mainly serves the elderly, and Medicaid helps low-income people. The characteristics of these groups may increase the medium- or long-term HCP, and the persistence of “dual-eligible” beneficiaries was higher (5). In addition to the characteristics of beneficiaries, the financial level and benefit package of an insurance service also affect the level of HCP. Although the NCMS in rural China has almost achieved full coverage, its reimbursement is limited by its deductible and reimbursement catalog. For some rural residents, the reimbursement is extremely small, particularly in cases with huge medical expenses. A small number of residents with poor economic conditions may abandon treatment or switch to palliative care at home because they are unable to afford further treatment. Another situation is that some residents with good economic conditions select hospitals outside of the county for better treatment at their own expense, which is also the neglected part of this study. The residents mentioned above show a transfer from high to low expenditure in the insurance database, but the “low expenditure” performance does not represent their actual medical demands and behaviors. These residents were included in the “transiently” high-cost group, which pulled the HCP down. Moreover, owing to the gap between the medical level of rural China and high-income countries, the poor prognoses of some complex and serious diseases may result in some high-cost patients entering a state of palliative care or death and finally being lost to follow-up in the cohort.

### Health status and family support are main drivers for high-cost persistence

Single-factor and binary logistic regression analyses showed that age, burden population ratio, subsistence allowance, and main disease were related to HCP. Age and main disease can be attributed to health status. Patients in the persistently high-cost cohort were older. This observation was the same as that of Cohen, who analyzed United States data from 2006 to 2007 (1). Elderly people are at risk of various diseases because of physiological function degradation and low self-resistance. They have higher demands for medical services and are prone to high-cost services. The disease prognoses of elderly residents are often poor, who are prone to chronic diseases and multiple complications. These are the possible reasons for the persistently high-cost status of elderly residents. In terms of disease factors, residents with malignant tumors and blood and immune diseases are prone to sustain high expenses, and this status may be related to the characteristics of diseases. Common blood and immune diseases include various kinds of anemia and rheumatic immune diseases, which often have the characteristics of protracted and lifelong medication and regular review. The incidence rates of malignant tumors have been increasing in recent years. Treatments for malignant tumors are characterized by High drug costs and frequent inpatient care. In summary, diseases that easily cause sustained high expenditure often have the characteristics of chronic and high medical service demand. At present, research on the relationship between diseases and HCP is limited, although some studies have shown that people suffering from a variety of chronic diseases and multi-system diseases are the key influencing factors of high-cost patients (19).

Burden population ratio and subsistence allowance can be attributed to family support. Few studies have shown a relationship between family support factors and HCP. In the present study, family structure was presented as the proportion of family burden population (burden population ratio), which has a high correlation. Compared with transiently high-cost patients' families, persistently high-cost patients' families have a larger proportion of the family burden population (the elderly and children). On average, two-thirds of persistently high-cost families' population is non-labor population, which is usually found in empty-nest families and Chinese left-behind families (Supplementary Table 1). These families often have higher medical needs, and when high expenses are incurred, weak family support enables these patients to go to hospitals in the county to receive medical and nursing services, especially inpatient services, resulting in the continuation of the high-cost state. Thus, for these structurally fragile families, targeted management is necessary. In addition to demography affecting family support, another important factor is economic conditions. Given that this study cannot incorporate economic factors, an alternative variable for subsistence allowance was added. Families with subsistence allowances are more likely to have persistently high medical expenses than families without low-income insurance, and this situation may be related to the fact that low-income families have a high medical demand. The reason is that residents with subsistence allowances usually partially or completely lose their ability to work because of illness or disability. Moreover, the welfare policy of second reimbursement (Supplementary Table 1) promotes transformation from medical demand into utilization, even if unreasonable. Determinants, such as gender, race, and marital status, have been explored. The analysis of statistical data in the United States from 2011 to 2012 has shown that women accounted for 56.3% of the population with persistently high-cost status, and this result is consistent with the results of the present study (20).

### Inpatient utilization as a mediator is a prominent manifestation of persistently high-cost patients

We found that the HCP is highly correlated with the previous utilization of inpatient services. Persistently high-cost patients had an average of 2.25 hospitalizations and 34.36 days hospital stay in the initial year, and the largest proportion of hospitalizations was found in county hospitals. The utilization of inpatient services plays a partial mediating role in the influence of family support and main disease on HCP. Families with fragile structures have higher rates of medical service utilization because of their members' poor physical conditions and the support of secondary reimbursement policy which promotes the conversion of their needs into utilization, especially inpatient utilization. Thus, these families are more likely to sustain high expenditures. The impacts of diseases on hospitalization are direct, such as malignant tumors, which require hospitalization. In addition, the service capacity of township health centers affects HCP through the utilization of inpatient services, and a positive correlation between the service capacity of township health centers and their inpatient service utilization was found. The reimbursement ratio of NCMS is higher in inpatients than in outpatients and higher in township health centers than in county hospitals. High-quality township health centers are likely to result in the transfer of patients to inpatient services because of the high reimbursement rates of primary medical insurance. This situation will lead to residents' unreasonable use of medical resources because of the preferential primary medical reimbursement system. However, this study is unable to determine whether residents' utilization is an overuse. High-cost patients often have the characteristic of excessive utilization of medical services. The evaluation of emergency services in nine countries including the United States has pointed out that the unreasonable rates of emergency and hospitalization are higher in high-cost patients than in ordinary patients (21). Moreover, a clear overutilization of some expensive medical services was observed. These unnecessary services may have been provided by primary care doctors at a relatively low cost. High-cost patients are prone to the overutilization of medical services, which is manifested in repeated visits, higher visits, and unreasonable admissions (22, 23). Further explaining the relationship between excessive service utilization and HCP is the direction of future research and improvement.

### Improve and use the patient information system to accurately identify and manage persistently high-cost patients

Heavy and long-term economic burden is imposed on persistently high-cost patients and also consumes a huge amount of health insurance funds. These patients should be included in the priority management of the regulatory department. This research determined the main characteristics of persistently high-cost patients, which included the elderly, people with fragile family structures and persistent diseases (mainly blood immune system diseases and malignant tumors), and high utilization of medical services, especially inpatient services. These indicators help us identify persistently high-cost patients, provided they are supported by a complete, interoperable, and efficient information system. At present, in China, there is a lack of an integrated database of individual health and socio-demographic information. Although residents have established health records in the community, information from these records is not comprehensive enough, and its use is limited. These situations decreased the precision of our management strategy for high-cost patients. In terms of management methods, Hong and his colleagues summarized the management experience of high-cost patients. Common methods include risk prediction software, chronic disease criteria, and physician assessment (24). Health Care Provider Alliance's Citywide Care Management System in Camden, NJ, identifies and targets high-cost patients through insurance claims, and obtains their consent to enroll in management programs by reaching targeted patients during admissions and emergency services (25). On the basis of continuous doctor-patient information exchange, we will consider the influencing factors of persistently high-cost patients in our planned targeted health service integration programs. Specific targets include persistently high-cost population marking, effective doctor-patient interaction, and continuous information exchange among multilevel institutions.

### Limitation

This study has several limitations. First, NCMS data limitations prevented the inclusion of the following persistently high-cost patients: (1) patients who were unable to afford medical expenses and gave up treatment or palliative care; (2) individuals who were able to pursue higher medical quality and selected out-of-county or private hospitals at their own expenses; and (3) deceased patients. These limitations may have led to the underestimation of HCP. Second, owing to the population-based data of the NCMS, including accurate variables, such as economic conditions, educational attainment, and occupation, was difficult, and the driving factors were limited. Nevertheless, we constructed some variables related to economic conditions, family structures, and social environment to compensate for the deficiency. Third, differences in HCP were found between years and showed a decreasing trend, which was probably related to underestimation. The reasons need to be further explored. Finally, this study did not analyze the rationality of healthcare utilization by persistently high-cost patients, which will be the focus of future research.

## Conclusion

High-cost patients in rural China were characterized by persistence, and the persistence rate was about 31.48% over a 2-year span. HCP decreased when the observation time interval was 3 years or more. Persistently high-cost patients were older and were much more likely to have malignant tumors and blood and immune diseases, which required regular examination and incurred high medication and hospitalization costs. Fragile family structure and the government's secondary reimbursement policy for low-income people both increased HCP. The expenditures of persistently high-cost patients were largely attributable to inpatient service utilization. Thus, improving patient information systems, monitoring potential persistently high-cost patients through risk factor labeling, and targeting their risk factors for early intervention are necessary.

## Data availability statement

The data analyzed in this study is subject to the following licenses/restrictions: The dataset is from the National Health Insurance database and is not publicly available due to administrative restrictions. Requests to access these datasets should be directed to YZ, yanzhang@hust.edu.cn.

## Author contributions

CW, SL, and YZ designed the study. YZ and SL acquired the data. CW conducted the data cleaning, statistical analysis, drafted the manuscript, and which all authors substantially reviewed and revised. All authors read and approved the final manuscript.

## Funding

This research was funded by the National Natural Science Foundation of China (Grant Nos: 71974064 and 72104086). The organization had no role in the study design, data collection, analysis,interpretation, and in writing the manuscript.

## Conflict of interest

The authors declare that the research was conducted in the absence of any commercial or financial relationships that could be construed as a potential conflict of interest.

## Publisher's note

All claims expressed in this article are solely those of the authors and do not necessarily represent those of their affiliated organizations, or those of the publisher, the editors and the reviewers. Any product that may be evaluated in this article, or claim that may be made by its manufacturer, is not guaranteed or endorsed by the publisher.

## Abbreviations

HCP: High-Cost Persistence
NCMS: New Rural Cooperative Medical Scheme
ICD-10: The 10th Revision of the International Classification of Diseases.
