# Supplementary material for: Drivers of high-cost persistence in rural China: A population-based retrospective study
Source: Front Public Health. 2022 Dec 6;10:988664. doi: 10.3389/fpubh.2022.988664 (PMC9763318; doi:10.3389/fpubh.2022.988664)
Supplement: Supplementary file 1 [file Data_Sheet_1.docx]

Supplementary Material

**Supplementary File 1** **Supplementary introduction of associated terms (Table S1)**

**Supplementary File 2** **List of ICD-10 codes used to identify main disease categories (Table S2)**

**Supplementary File 3 Definitions and categories of factors (Table S3)**

**Supplementary File 1**

**Table S1 Supplementary introduction of associated terms**

| **Terms** | **Introduction** |
| --- | --- |
| Supplementary Medical Insurance for Major Illnesses | Supplementary Medical Insurance for Major Illnesses, which is issued by the Chinese government and implemented nationwide in 2015, is a further compensation for the high Medical expenses incurred by patients with specific Major diseases. |
| Healthy Targeted Poverty Alleviation Policy | Healthy Targeted Poverty Alleviation Policy, promoted by the Chinese government in 2016, focuses on the poverty-stricken population, achieving full coverage of basic medical insurance and basic medical treatment. |
| Empty-Nest Family | Empty-Nest Family is a kind of family in which the elderly are left living alone without their children in the family home. |
| Chinese Left-Behind Family | Chinese Left-Behind Family refers to families in which both parents are rural-to-urban migrant workers while their children remain in rural regions under the care of grandparents. |
| Second Reimbursement Policy | Secondary Reimbursement Policy is a major illness relief policy for subsistence allowance households and marginal households. Local governments pay for the secondary reimbursement for out-of-pocket expenses of these residents. |

**Supplementary File 2**

**Table S2 List of ICD-10 codes used to identify main disease categories**

| **Category** | **Inclusions for ICD-10 codes** |
| --- | --- |
| Infectious and parasitic | A00~B99 |
| Tumor | C00~D48 |
| Blood and immune | D50~D89 |
| Endocrine and metabolic | E00~E90 |
| Nervous system and mental | F00~G99 |
| Circulatory system | I00~I99 |
| Respiratory system | J00~J99 |
| Genitourinary system | N00~N99 |
| Others | H00~H95, K00~L99, M00~M99, O00~U99, and no encoded fields |

**Supplementary File 3**

**Table S3 Definitions and categories of associated factors**

| **Factors** | **Definitions and categories** |
| --- | --- |
| ***Socio-demographic characteristics*** | |
| Age | Age of the patient  0=≤5  1=6~18  2=19~40  3=41~59  4=≥60 |
| Gender | Gender of the patient  0=male, 1=female |
| Burden population ratio | Non-labor population divided by the total family population (continuous: 0~1) |
| Subsistence allowance | Whether or not the patient is living with subsistence allowance  0=No, 1=yes |
| ***Socio-environmental characteristics*** | |
| Terrain | Terrain type of residential area  0=Plain  1=Downland  2=Hill  3=Mountain |
| Distance to county hospital | Transportation distance from the residence to the county hospital (Google Map, km) |
| Township health center service capacity | Evaluation standard: National Health Commission of the PRC.  Service capacity evaluation guide for township health centers; 2019. Available from: [http://www.nhc.gov.cn/jws/s2908/201904/523e5775cdba451a81ab2fbc0628d9f0/files/c31bea99be2f4d62b9b6934c1c5cbc49.pdf.](http://www.nhc.gov.cn/mohwsbwstjxxzx/s7967/201306/fe0b764da4f74b858eb55264572eab92.shtml.) Accessed November 2, 2022.  Dimensions: (1) Function and resource configuration (11 items). (2) Basic medical and public health services (36 items). (3) Operational control (40 items). (4) Integrated management (13 items).  Evaluation results: A, B, C & D. The results were obtained from the Dangyang Municipal Health Commission. The evaluation results of Dangyang City are divided into three levels: A, B & C, and none of the township health centers had a D result. |
| ***Disease*** | |
| Main disease | Principal diagnosis (ICD-10) of high-cost patients in the NCMS database in 2016  0=Others  1=Infectious and parasitic  2=Malignant tumor  3=Blood and immune  4=Endocrine and metabolic  5=Nervous system and mental  6=Circulatory system  7=Respiratory system  8=Genitourinary system |
| ***Health care utilization*** | |
| Hospitalization frequency | Hospitalization frequency of the patient during 2016 |
| Hospitalizations frequency at different levels of institutions | 1=Hospitalization frequency in township Health Center of the patient during 2016  2=Hospitalization frequency in county hospital of the patient during 2016  3=Hospitalization frequency outside of the county of the patient during 2016 |
| Outpatient visits | Outpatient visits of the patient during 2016 |
| Length of stay | Total length of stay during 2016 (day) |
